# Probability of pharmacokinetic/pharmacodynamic target attainment for different piperacillin/tazobactam dosing regimens in renally impaired patients in a non‐intensive care unit setting

**DOI:** 10.1002/bcp.70153

**Published:** 2025-06-29

**Authors:** Emma Dohmann, Stefan Hagel, Max Kurlbaum, Paul Schellong, Oliver Scherf‐Clavel, Güzin Surat

**Affiliations:** ^1^ Julius‐Maximilians‐Universität Würzburg Würzburg Germany; ^2^ Institute for Infectious Diseases and Infection Control Jena University Hospital‐Friedrich Schiller University Jena Jena Germany; ^3^ Department of Internal Medicine I, Division of Endocrinology and Diabetes University Hospital Würzburg Würzburg Germany; ^4^ Central Laboratory, Core Unit Clinical Mass Spectrometry University Hospital Würzburg Würzburg Germany; ^5^ Ludwig‐Maximilians‐Universität München, Department of Pharmacy Munich Germany; ^6^ Unit for Infection Control and Antimicrobial Stewardship University Hospital Würzburg Würzburg Germany

**Keywords:** haemodialysis, piperacillin/tazobactam, *Pseudomonas aeruginosa*, susceptible increased exposure (I), therapeutic drug monitoring

## Abstract

**Aims:**

To optimize antibiotic therapy for pathogens classified as *susceptible, increased exposure (I)*, an increased exposure of piperacillin/tazobactam (PTZ) is required. However, dosing recommendations are currently only available for patients with normal renal function. The aim of the study was to assess whether the recommended dosages of PTZ for patients with impaired renal function achieve adequate pharmacokinetic/pharmacodynamic (PK/PD) targets for pathogens classified as *susceptible, increased exposure (I)*.

**Methods:**

Overall, 49 patients with impaired renal function were included in this study (estimated glomerular filtration rate [eGFR] 20–40 mL/min: *n =* 20; eGFR <20 mL/min: *n =* 19; intermittent haemodialysis: *n =* 10). Peak, trough and between‐dosing interval piperacillin concentrations were determined. The primary endpoint of the study was the probability of target attainment (PTA) for a conservative (fT 60% > minimal inhibitory concentration) and an aggressive PK/PD target (fT 100% > 4× minimal inhibitory concentration). First, a population pharmacokinetic model was developed followed by a model‐based simulation.

**Results:**

For the conservative PK/PD target, a PTA of >90% is achieved in all patients receiving intermittent short infusions, following the dosing recommendations outlined in the Summary of Product Characteristics (SmPC). For the more aggressive target, the PTA was <15% across all groups when using short infusions with SmPC dosing. Only continuous infusion with an increased daily dose in patients with eGFRs of 30 and 40 mL/min achieved a PTA of >90% in all patients.

**Conclusions:**

Dosing according to the SmPC is only sufficient to achieve a conservative PK/PD target. In comparison, simulating an increased dosing with continuous administration attained an aggressive PK/PD target. Offering alternative dosing regimens may be of interest for severe infections with difficult to treat foci caused by *Pseudomonas aeruginosa* even in non‐intensive care unit patients.

**What is known already about this subject**

- The literature around therapeutic drug monitoring on β‐lactam antibiotics is complex but, owing to the attribute of time‐dependent bacterial killing, at least prolonged infusion is favoured.
- The best predictor is the time of the free drug concentration exceeding the minimal inhibitory concentration during dosage interval.
- There is meta‐analysis suggesting reduced mortality in patients receiving prolonged or continuous infusion *vs*. intermittent infusion.

**What the study adds**

- The study focuses on the modified dosing recommendation of the European Society of Clinical Microbiology and Infectious Diseases, when increased exposure of piperacillin/tazobactam is required by the respective susceptibly testing on *Pseudomonas aeruginosa*.
- The study encompasses different dosing strategies (intermittent, prolonged, continuous) in different renal settings using different model‐underlined therapeutic drug monitoring targets based on different clinical backgrounds given increased exposure of piperacillin is called for.

## 1 INTRODUCTION

Piperacillin/tazobactam (PTZ) is a broad‐spectrum antibiotic effective against pathogens such as *Pseudomonas aeruginosa*. It is commonly used to treat pneumonia, intra‐abdominal infections, complicated urinary tract infections, as well as febrile neutropenia and sepsis.^1^, ^2^ Piperacillin is primarily eliminated by the kidneys through glomerular filtration and tubular secretion, making its clearance highly sensitive to changes in kidney function.^3^, ^4^, ^5^ The European Committee on Antimicrobial Susceptibility Testing (EUCAST) provides dosing recommendations for PTZ based on whether the pathogen is classified as *susceptible, standard dose* (labelled *S*) or *susceptible, increased exposure* (labelled *I*).^6^ This distinction accounts for the need for higher.

Drug exposure in cases of reduced wild‐type pathogen susceptibility, acquired resistance, or inherently less susceptible pathogens (e.g., *P. aeruginosa*). However, EUCAST's dosing recommendations are only provided for patients with normal renal function without any guidance on dosing in patients with impaired renal function. The Summary of Product Characteristics (SmPC) for PTZ suggests a maximum dose of 4.5 g every 8 h in patients with a creatinine clearance of 20–40 mL/min, or every 12 h in patients with a creatinine clearance of <20 mL/min. The aim of the study was to determine whether the recommended dosages in the SmPC for patients with impaired renal function achieve the necessary pharmacokinetic (PK)/pharmacodynamic (PD) targets for pathogens tested as *susceptible, increased exposure (I)*, respectively to identify the dosage regimens required to meet these targets.

In this study, we compared a conservative PK/PD target of 60% of the time > minimal inhibitory concentration (MIC) to a more aggressive PK/PD target of 100% of the time >4× MIC.

## 2 METHODS

### 2.1 Study design

This prospective multicentre study, conducted between 2 March 2022 and 17 March 2023, included 49 patients from general wards—30 from Würzburg University Hospital and 19 from Jena University Hospital—all receiving intermittent intravenous infusions of PTZ (4000 mg/500 mg). Inclusion criteria were: (i) intermittent infusion of PTZ; (ii) chronic kidney disease with an estimated glomerular filtration rate (eGFR) of <40 mL/min or intermittent haemodialysis (iHD) 3‐times weekly schedule; and (iii) informed consent. Exclusion criteria were: (i) pregnancy or breastfeeding; (ii) age 18 years; (iii) hypersensitivity against β‐lactams; and (iv) participation in other clinical trials. The patients were divided into 3 groups based on their renal function (group 1: eGFR 20–40 mL/min, group 2: eGFR <20 mL/min, group 3: patients receiving haemodialysis 3 times a week). Demographic data, including age, height and weight, along with clinical data and antibiotic dosing information (regimens, infusion durations, sampling times and therapy timing), were obtained from electronic patient records and are presented in Table 1. The eGFR was calculated with the Modification of Diet in Renal Disease formula (mL/min/1.73m^2^). The ethics committees of the University of Würzburg and Jena have reviewed and approved the study (approval number: 110/21 and 2021–2399).

**TABLE 1:** Patients' characteristics.

	Total	Group 1 eGFR 20–40 mL/min	Group 2 eGFR <20 mL/min	Group 3 haemodialysis
Number of patients (%)	49	20 (40.8)	19 (38.8)	10 (20.4)
Hospital				
Würzburg (%)	30 (61.2)	8	12	10
Jena (%)	19 (38.8)	12	7	
Sex				
Male (%)	31 (63.3)	11	12	8
Female (%)	18 (36.7)	9	7	2
Age (years)	69.3 ± 11.1	71.8 ± 9.3	68.8 ± 10.4	65.2 ± 14.7
Height (cm)	170.9 ± 8.9	169.3 ± 9.1	171.5 ± 8.2	173.1 ± 10.1
Weight (kg)	76.1 ± 17.1	75.7 ± 17.2	75.1 ± 16.6	78.9 ± 19.2
BMI (kg/m^**2**^)	26.1 ± 5.6	26.4 ± 5.9	25.5 ± 5.3	26.3 ± 5.8
eGFR (mL/min/1.73m^**2**^)	19.9 ± 9.3	29.2 ± 6.6	14.6 ± 3.6	11.2 ± 3.3
Time of dialysis (min)				258.8 ± 24.8
Recommended dosing Regimen according to the SmPC		4.5 g q8 h	4.5 g q12 h	4.5 g q12 h + 2.25 g after haemodialysis

*Note*: Data reported as total number (%) or mean ± standard deviation; eGFR is calculated with Modification of Diet in Renal Disease in Würzburg and CKD‐EPI in Jena.

Abbreviations: BMI, body mass index; eGFR, estimated glomerular filtration rate.

### 2.2 PK/PD targets

The conservative PK/PD target attainment was defined as free piperacillin serum concentration >16 mg/L for at least 60% of the time. This concentration corresponds to the MIC breakpoint of *P. aeruginosa* (R > 16 mg/L), determined by EUCAST.^6^ The aggressive PK/PD target attainment was set at free piperacillin serum concentration >4× MIC (64 mg/L) for 100% of the time. This target corresponds to recently published recommendations, which suggest free plasma β‐lactam concentrations to be >4–8× MIC of the tested bacteria for 100% of the time for intensive care unit patients.^7^

### 2.3 PTZ administration

PTZ was administered according to the discretion of the treating physician and was administered in almost all patients according to SmPC recommendations: patients with eGFR 20–40 mL/min received 3 daily doses of 4.5 g PTZ over 30 min (group 1); those with eGFR <20 mL/min received 2 daily doses of 4.5 g (group 2); and patients undergoing haemodialysis also received 2 daily doses of 4.5 g (group 3). Of the patients undergoing iHD, 3 out of 10 received their last PTZ infusion within 1.5 h before dialysis, while 7 received their morning infusion immediately after dialysis (data not shown). There were different indications for which patients received PTZ treatment: patients with nosocomial infections with unclear focus received empirical treatment with PTZ, while patients with hospital acquired pneumonia or severe skin infections, with or without bacteraemia, received targeted therapy with PTZ.

### 2.4 Sample collection and piperacillin concentration measurement

To make sure that PTZ was in steady state, blood samples were collected after 3 days of PTZ therapy at 3 time points: within 30 min before start of the PTZ infusion, within 30 min after the end of infusion, and between dosing intervals (either 4 or 6 h after infusion) for groups 1 and 2. Blood samples from group 3 were collected immediately before and after an iHD session. The blood samples were transported directly to the central laboratory, centrifuged at 3326 *g* for 10 min at 20°C, and then stored at −80°C within 2 h of collection after removing the supernatant.

The serum concentration of piperacillin in all blood samples from Würzburg and Jena was measured at the central laboratory in Würzburg. The serum concentration of piperacillin was determined by high performance liquid chromatography–tandem mass spectrometry using a QTRAP 4500MD (Sciex, Framingham, MA, USA) and an Agilent 1290 UHPLC system (Agilent, Waldbronn, Germany) at the Würzburg University Hospital's central laboratory. Separation was performed by a XBridge BEH C18 2.5 μm 3.0 × 75 mm (Waters, Eschborn, Germany) column including online SPE (Oasis HLB Column 15 μm (2.1 × 20 mm, Waters, Eschborn, Germany). Mobile phases consisted of (A) water (0.1% sodium acetate [pH 3.8]) and phase (B) methanol. All chemicals and reagents were supplied by Sigma Aldrich; Piperacillin‐d5 was supplied by TRC chemicals (Toronto, Canada). A 190‐μL aliquot of serum was mixed with 30 μL of internal standard solution (Piperacillin‐d5) and vortexed for 10 s followed by 10 min of incubation. After dilution with 800 μL of acetonitrile the samples were vortexed (30 s) and centrifuged (15 000 *g*) for 5 min. A 100‐μL aliquot of the supernatant were diluted with 300 μL of mobile phase A and vortexed again (10 s). After 10 min of incubation samples were centrifuged again (5 min, 15 000 *g*) and 150 μL of supernatant were transferred into 96‐well plates, 15 μL were used for liquid chromatography–mass spectrometry measurement. Quantitation was performed using linear regression with 1/x weighting based on ratios of analyte and corresponding isotope labelled standard. Analytical range was from 0.5 to 190.0 mg/L. Samples with results above the upper limit of quantitation were diluted and re‐analysed. The results of diluted samples were used for further analysis. Even though PTZ was administered, only the concentration of piperacillin was determined. The method was validated according to European Medicines Agency guidelines on bioanalytical method validation.^8^ A free plasma concentration of 16 mg/L is equivalent to a total plasma concentration of 19.8 mg/L for piperacillin. Piperacillin plasma concentrations are presented as total concentrations.

### 2.5 Population PK model

Overall, 80 samples from 30 patients from the Würzburg University Hospital (one concentration was excluded, due to implausibly high concentration, probably due to sample collection error) and 55 samples from 19 patients from the Jena University Hospital (one concentration was excluded due to implausible high concentration, probably due to erroneous sample collection) were included in the analysis. A population PK model was developed using Monolix 2023R1 (Lixoft, Antony, France). A 1 compartment model with linear elimination was selected as the starting point. Patients with iHD were modelled by including a second clearance process during the times of dialysis. The dialysis clearance was turned on or off by a regressor variable (θ_HD_ = 0 or 1) according to the documented dialysis times. Available covariates for modelling were age, body mass index (BMI), body surface area (BSA, Mosteller formula), height, weight, eGFR (Modification of Diet in Renal Disease formula individualized using BSA) and sex. Continuous covariates were tested in a power law model if they showed significant correlation with the individual random effects (5 random samples from the conditional distribution per patient):

$${\mathrm{CL}}_{\mathrm{ind}}={\mathrm{CL}}_{\mathrm{pop}}\cdot {\left(\frac{\text{eGFR}}{21.8\;\mathrm{mL}/\min}\right)}^{\beta_{\text{eGFR}}}\cdot e^{\eta_{\mathrm{CL}}}+\left({\mathrm{CL}}_{\mathrm{HD}}\cdot \theta_{\mathrm{HD}}\right)$$

$${\mathrm{Vd}}_{\mathrm{ind}}={\mathrm{Vd}}_{\mathrm{pop}}\cdot {\left(\frac{\mathrm{BSA}}{1.66\;m2}\right)}^{\beta_{\mathrm{BSA}}}\cdot e^{\eta_{\mathrm{Vd}}}$$

Categorical covariates (i.e.) sex was modelled as follows:

${\mathrm{Vd}}_{\mathrm{ind}}={\mathrm{Vd}}_{\mathrm{pop}}\cdot e^{\beta_{\mathrm{sex}}\cdot \theta_{\mathrm{sex}}}\cdot e^{\eta_{\mathrm{Vd}}}$ with $\theta_{\mathrm{sex}}=1$ for female and $\theta_{\mathrm{sex}}=0$ for male patients.

BSA, sex and eGFR were tested as covariates on CL, whereas BMI, weight, height, sex and BSA were considered as covariates on Vd (Table S1 and Figure S4). A covariate effect (β_eGFR_, β_BSA_) was considered statistically significant if the Wald test from stochastic approximation yielded a *P*‐value of ≤.01 and the forward inclusion led to a significant reduction of the objective function value (*P* ≤ .05) and backwards elimination significantly increased the objective function value (*P* ≤ .01).

### 2.6 Monte Carlo simulations to assess probability of target attainment

The conservative PK/PD target was set at an unbound concentration above 16 mg/L for ≥60% of the time during a dosing interval, while the aggressive PK/PD target was set to an unbound concentration above 4× MIC (MIC = 16 mg/L) for 100% of the time during a dosing interval.

The probability of target attainment (PTA) was calculated for 4 groups of patients for the conservative PK/PD target: eGFR 40, 30, 20 mL/min without haemodialysis; and eGFR 10 mL/min with iHD (once daily for 4 h directly after piperacillin infusion ended), respectively. For each group different strategies of administration were evaluated: intermittent infusion of 4.5 g PTZ every (q)12 h over a 30‐min infusion; prolonged infusion of 4.5 g PTZ q12 h over 3 h infusion duration; and prolonged infusion of 2.25 g PTZ q8 h over 3 h infusion duration. To evaluate appropriate dosing strategies for patients with an eGFR between 20 and 40 mL/min, 4.5 g PTZ q8 h and q12 h were simulated as short infusion over 30 min and as prolonged infusion over 3 h. For the more aggressive PK/PD target, PTA was evaluated for 5 groups of patients: eGFR 40, 30, 20 and 10 mL/min, with and without haemodialysis. The strategies of administration that were tested included a 30‐min short infusion (dosing according to SmPC), a 4‐h prolonged infusion with increased dosing (eGFR 40 mL/min with 8000 mg piperacillin q8 h, eGFR 30 mL/min with 8000 mg q8 h, eGFR 20 mL/min with 8000 mg q8 h, eGFR 10 mL/min without haemodialysis with 6000 mg q8 h and eGFR 10 mL/min with haemodialysis with 6000 mg q12 h plus an additional 6000 mg over 4 h during haemodialysis), and continuous infusions (eGFR 40 mL/min with 16 000 mg q24 h, eGFR 30 mL/min with 16 000 mg q24 h, eGFR 20 mL/min with 12 000 mg q24 h, eGFR 10 mL/min without haemodialysis with 8000 mg q24 h and eGFR 10 mL/min with haemodialysis with 8000 mg q24 h plus an additional 2000 mg over 4 h during haemodialysis).

First, a population PK model was developed and then a model‐based simulation was performed.

Ten in silico trials were performed with 100 virtual patients per dosing group and per trial. The sources of variability included the modelled IIV, uncertainty of parameter estimation and the covariate BSA was set to be drawn from a lognormal distribution with mean 1.88 m^2^ and a standard deviation (SD) of 0.12 m^2^. The eGFR was set exactly to the value according to the group. The achievement of the PK/PD target was calculated from a dummy variable that was set to either 0 or 1 if the simulated concentration was below or above the target concentration for every simulated time point (0.1 h intervals) during the runtime of the simulation. After that, the dummy variable was integrated over time and target attainment (either 60 or 100% of the dosage interval above target) was calculated from the integral.

All simulations were performed in Simulx2023R1 (Lixoft, Antony, France) and the results were visualized using R statistical software 4.0.5 (R Foundation for Statistical Computing, Vienna, Austria) using the *ggplot2* package (Hadley Wickham, Springer‐Verlag New York).

Since the developed popPK model simulates total concentrations, an unbound fraction of 80% found in the literature was used to estimate unbound piperacillin concentration by multiplication of simulated total concentration by 0.8.

## 3 RESULTS

### 3.1 Piperacillin concentration

In group 1 (eGFR 20–40 mL/min), the mean trough concentration of total piperacillin was 27.8 mg/L (*n =* 20, SD 35.9 mg/L), with a minimum of 2.5 mg/L and a maximum of 142 mg/L. The mean peak concentration in this group was 287.1 mg/L (*n =* 18, SD 101.9 mg/L), ranging from 40.3 to 447 mg/L. The mid dosing concentration was 74.6 mg/L (*n =* 18, SD 33.5 mg/L), with values from 20.8 to 157 mg/L. The dosage interval was aligned with the standard regimen dosing intervals for 14 patients. Six patients received PTZ at a q12 h interval instead of the recommended q8 h interval. It should be noted that some of these patients had a daily fluctuating eGFR close to 20 mL/min, which is the limit under which a q12 h dosing interval is recommended.

In group 2 (eGFR <20 mL/min), the mean trough concentration was 58.2 mg/L (*n =* 19, SD 57.9 mg/L), with a range of 6.4–179 mg/L. The mean peak concentration in this group was 282.6 mg/L (*n =* 19, SD 102 mg/L), with values from 136 to 515 mg/L. The mean interval concentration for group 2 was 97 mg/L (*n =* 19, SD 54.2 mg/L), with a range of 28.3 to 277 mg/L. The dosage interval was aligned with the standard regimen dosing intervals for 16 patients. Three patients received PTZ at a q8‐h interval instead of the recommended q12‐h interval. It should be noted that some of these patients had an eGFR close to 20 mL/min, above which a q8‐h interval is correct.

In group 3 (iHD) the mean piperacillin concentration before haemodialysis was 136.3 mg/L (*n =* 10, SD 136.7 mg/L), ranging from 6.3 to 403 mg/L. The mean concentration after haemodialysis was 22 mg/L (*n =* 10, SD 21.8 mg/L), with a minimum of 1.3 mg/L and a maximum of 60 mg/L. The dosage interval was according to the SmPC for 9 patients and 1 patient received PTZ in a q24‐h interval, instead of the recommended q12‐h interval.

### 3.2 Development of the PK model

Based on the available data, a 1‐compartment model was chosen. There was neither sufficient data to fit a more complex 2‐compartment model nor did the 1‐compartment model show any obvious systematic bias that would result from model misspecification. Thus, a 1‐compartment model was chosen as fit for the intended purpose. The model was parameterized with clearance (CL) and volume of distribution (Vd). Different residual error models were evaluated, and the proportional error model was chosen, as it led to no obvious pattern in the residuals (see Figures S1 and S2). In the stepwise testing of covariates, only BSA and eGFR were significant covariates. eGFR was a covariate on nondialysis clearance (= CL_pop_) and BSA on Vd (= Vd_pop_).

**FIGURE 1:**
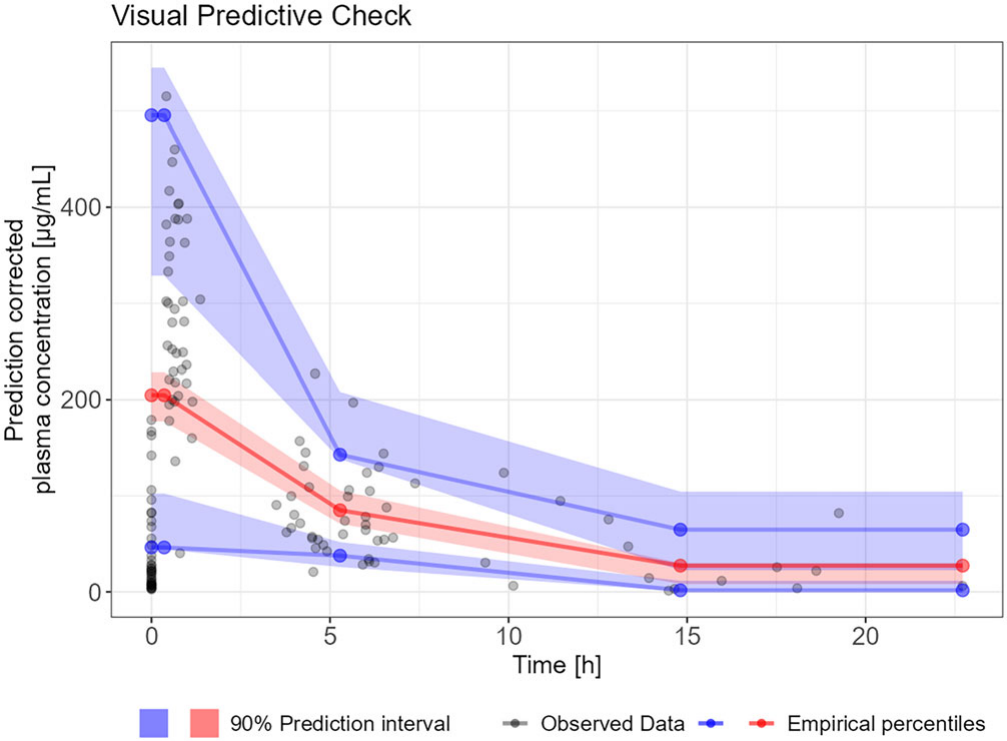
Visual predictive check. The solid lines represent the 5th (lower blue), 50th (red) and 95th (upper blue) percentiles of the observed data. Shaded regions represent the 90% confidence intervals surrounding the 5th, 50th and 95th percentiles from the predicted data. Overall, the plot demonstrates that the model predictions captured most observed piperacillin concentrations within the 5th and 95th percentiles of the simulated values.

The visual predictive check (Figure 1) and other goodness‐of‐fits plots (see Figures S1–S3) were acceptable. Population parameters could be estimated with good precision (relative standard error < 10%) except for haemodialysis clearance (probably due to the low number of patients). All other parameters could be estimated with acceptable precision (see Table 2). Interindividual variability on haemodialysis clearance was not included in the model, since the estimate was unreliable due to high standard error, which was also probably because of the small patient number.

**TABLE 2:** Population parameter estimates of the final model.

Parameter	Value	Standard error^1^	Relative standard error (%)
Vd_pop_ (L)	15.42	1.08	7.02
β_BSA_	1.46	0.42	28.7
CL_pop_ (L/h)	3.52	0.2	5.68
β_eGFR_	0.54	0.11	20.0
CL_HD_ (L/h)	3.96	1.36	34.4
ω_Vd_	0.24	0.056	23.2
ω_CL_	0.34	0.042	12.4
Proportional error	0.26	0.024	9.38

*Note*: β_BSA_, covariate effect of body surface area on Vd_pop_; β_eGFR_, covariate effect of estimated glomerular filtration rate on CL_pop_; CL_HD_, haemodialysis clearance; CL_pop_, population value for clearance; ω_CL_, standard deviation of random effects of CL_pop_; ω_Vd_, standard deviation of random effects of Vd_pop._^1^ calculated from the Fisher Information matrix obtained from stochastic approximation.

### 3.3 Monte Carlo simulation of PK/PD‐target attainment^9^

**FIGURE 2:**
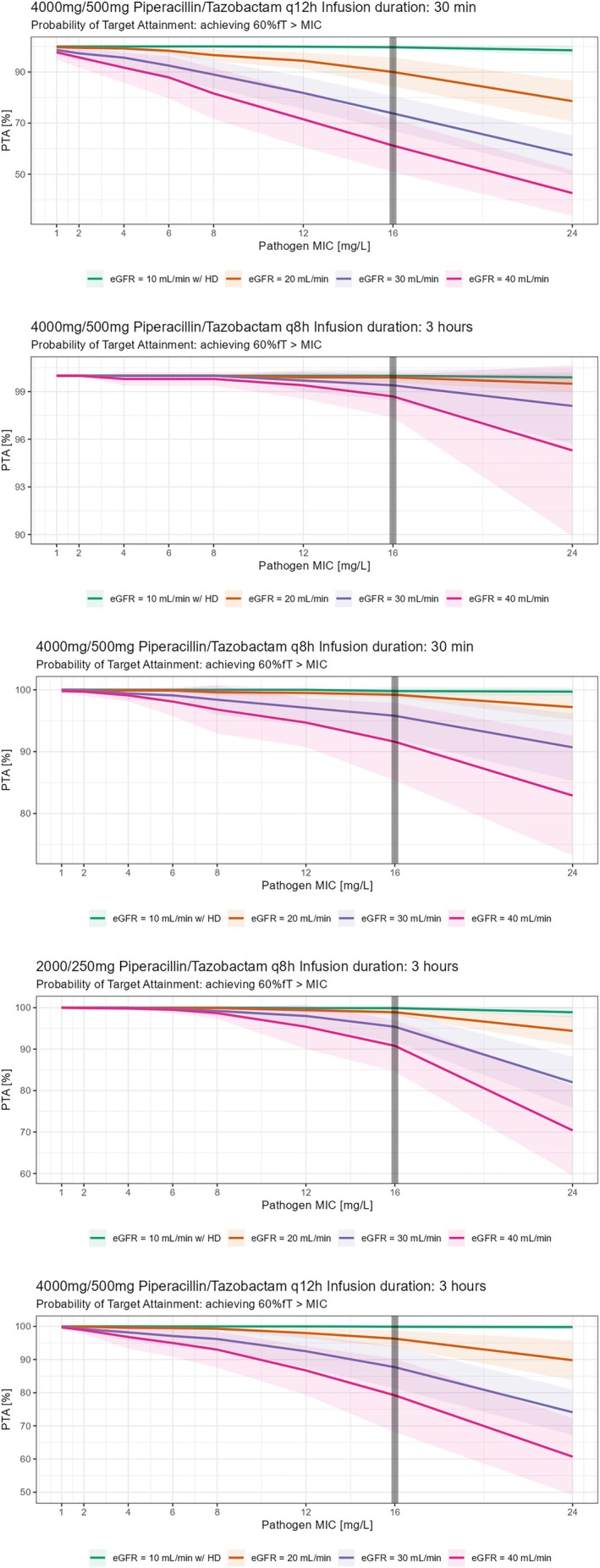
Probability of target attainment (PTA; conservative pharmacokinetic/pharmacodynamic target) in relation to desired unbound piperacillin concentration with dosing according to SmPC recommendations obtained from Monte Carlo simulations. The solid lines represent the mean of 10 virtual studies with 100 virtual patients per dosing group each and the shaded area indicates ±1 standard deviation. MIC, minimal inhibitory concentration.

**FIGURE 3:**
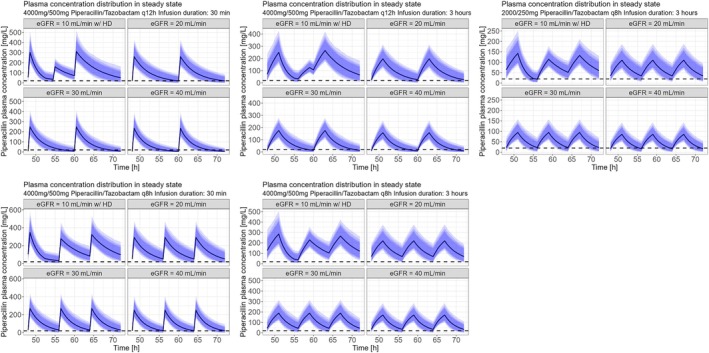
Plasma concentrations *vs.* time curves including prediction intervals from Monte Carlo simulations for different dosing strategies (conservative pharmacokinetic/pharmacodynamic target). The dashed line is the minimal inhibitory concentration (MIC) breakpoint of *Pseudomonas aeruginosa* (19.8 mg/L equivalent to 16 mg/L unbound concentration). Patients on haemodialysis (HD) received a bolus dose of 2000 mg/250 mg PTZ after HD if on a q12‐h regimen.

**FIGURE 4:**
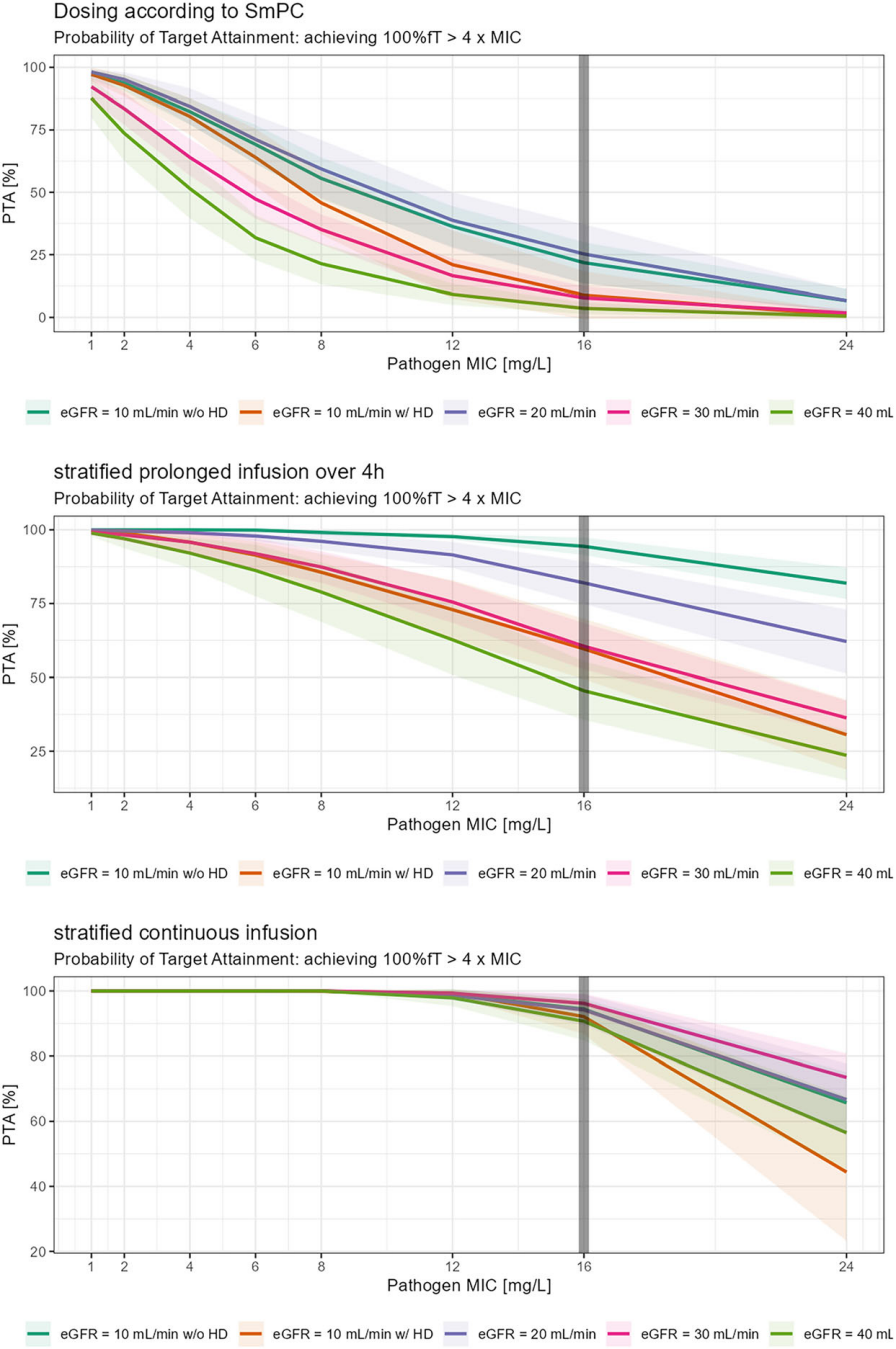
Probability of target attainment (PTA; aggressive pharmacokinetic/pharmacodynamic target) in relation to desired unbound piperacillin concentration obtained from Monte Carlo simulations. Prolonged 4‐h infusions with increased dosing as displayed above. The solid lines represent the mean of 10 virtual studies with 100 virtual patients per dosing group each and the shaded area indicates ±1 standard deviation.

**FIGURE 5:**
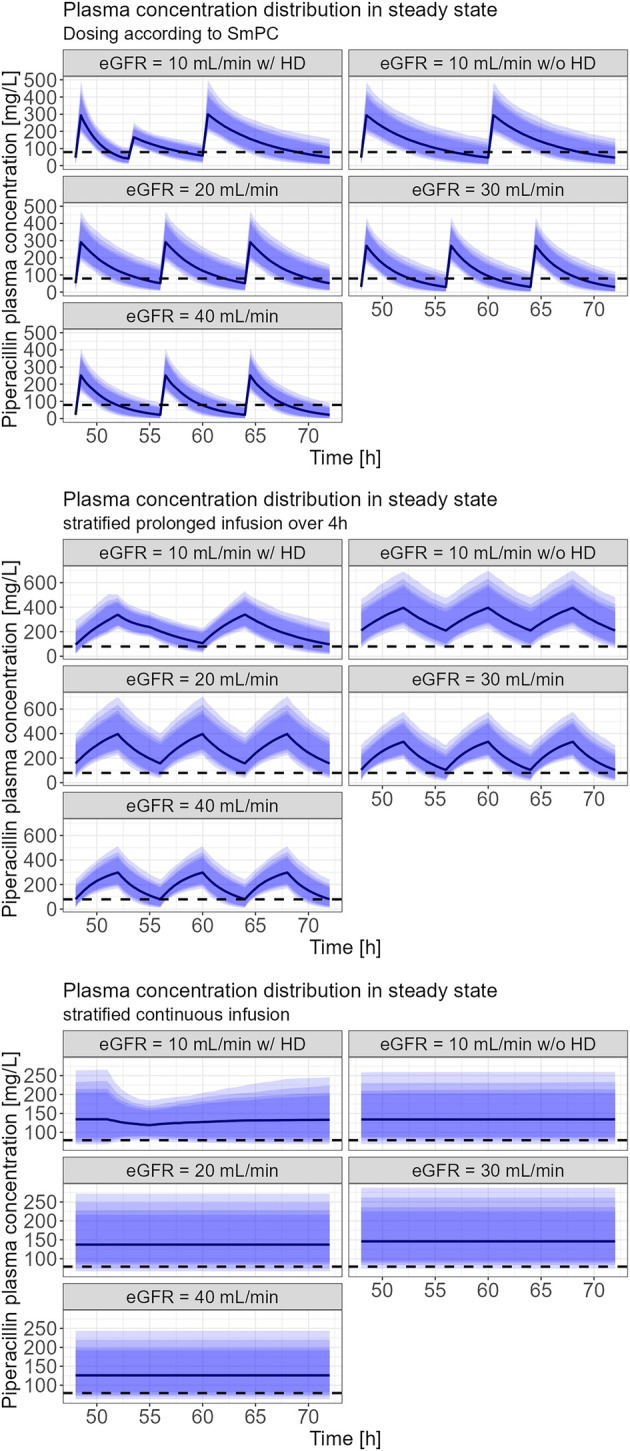
Piperacillin plasma concentration in relation to time (aggressive pharmacokinetic/pharmacodynamic target). Dashed line is the total plasma concentration that is equivalent to a 4‐fold minimal inhibitory concentration for pathogens classified with minimal inhibitory concentration of 16 mg/L (79.2 mg/L).

All planned simulations could be run and analysed (see Figure 2, 3, 4 and 5). For the *conservative* PK/PD target, patients with an eGFR between 20 and 40 mL/min receiving the recommended dosing of PTZ (4.5 g, q8 h, 30 min) resulted in a PTA of 99.2% (20 mL/min), 95.8% (30 mL/min) and 91.6% (40 mL/min) respectively. Prolongation of infusion over 3 h increased PTA in all patients to 100, 99.9, 99.4 and 98.7% respectively. In patients with haemodialysis the recommended dosing of PTZ (4.5 g, q12 h, 30 min) resulted in a PTA of 99.7%. Again, prolonged infusion (4.5 g, q12 h, 3 h) increased PTA to 99.9% in this group of patients (Table 3). For the *aggressive* PK/PD target, patients with eGFR 40 mL/min reached a PTA of 3.6% with dosing according to SmPC, a PTA of 45.5% with a 4‐h prolonged infusion, and a PTA of 90.7% with continuous infusion. Patients with eGFR 30 mL/min reached a PTA of 7.8% with dosing according to SmPC, a PTA of 60.5% with a 4‐h prolonged infusion, and a PTA of 96.2% with continuous infusion. Patients with eGFR 20 mL/min reached a PTA of 25.3% with dosing according to SmPC, a PTA of 82% with a 4‐h prolonged infusion, and a PTA of 94.2% with continuous infusion. Patients with eGFR of 10 mL/min with haemodialysis reached a PTA of 8.9% with dosing according to SmPC, a PTA of 59.6% with a 4‐h prolonged infusion, and a PTA of 92.1% with continuous infusion. Patients with eGFR 10 mL/min without haemodialysis reached a PTA of 21.8% with dosing according to SmPC, a PTA of 94.4% with a 4‐h prolonged infusion, and a PTA of 94.4% with continuous infusions (table 4). Not all PTA results shown in Tables 3 and 4 are mentioned.

**TABLE 3:** Conservative pharmacokinetic/pharmacodynamic target (fT > 60% > minimal inhibitory concentration).

	4.5 g q8 h over 30 min	4.5 g q8 h over 3 h	4.5 g q12 h over 30 min	4.5 g q12 h over3 h
eGFR 40 mL/min	91.6%	98.7%	61.2%	79.2%
eGFR 30 mL/min	95.8%	99.4%	73.8%	87.7%
eGFR 20 mL/min	99.2%	99.9%	90%	96.9%
eGFR 10 mL/min w/ HD	99.8%	100%	99.7%	99.9%

eGFR, estimated glomerular filtration rate; HD, haemodialysis.

**TABLE 4:** Aggressive pharmacokinetic/pharmacodynamic target (fT 100% > 4× minimal inhibitory concentration).

	Dosing according to SmPC	Stratified prolonged infusion over 4 h	Stratified continuous infusion
eGFR 40 mL/min	3.6%	45.4%	90.7%
eGFR 30 mL/min	7.8%	60.5%	96.2%
eGFR 20 mL/min	25.3%	82%	94.2%
eGFR 10 mL/min w/ HD	8.9%	59.6%	92.1%
eGFR 10 mL/min w/o HD	21.8%	94.4%	94.4

eGFR, estimated glomerular filtration rate; HD, haemodialysis; SmPC, Summary of Product Characteristics.

## 4 DISCUSSION

This study was initiated in light of modified definitions of susceptibility testing categories by EUCAST in 2019.^10^, ^11^ This change had not only wide implications for European microbiological laboratories but left question marks especially for the clinical workflow in antimicrobial stewardship programmes, for example, when giving advice on dosing in patients with renal impairment.^12^

This project shall help to guide PTZ dosing in patients with renal impairment when microbiology susceptibility reports indicate *high(er) dosage*. Our results show that intermittent or prolonged application of PTZ with a conservative PK/PD target achieves an adequate PTA, in accordance with SmPC recommendations too. However, the more aggressive PK/PD target, if aimed, can only be achieved through continuous application and an increased dosage for patients with eGFRs of 30 and 40 mL/min. Additionally, we observed significant variability in plasma piperacillin concentrations, with some patients experiencing sub‐ or supratherapeutic levels. In contrast, Zander *et al*. demonstrated that 100% of the patients receiving PTZ only twice daily due to renal impairment (creatinine clearance: 6–19 mL/min) achieved 100% fT > MIC (22.5 mg/L) on day 1 when administered 2 doses of 4.5 g PTZ over 30 min daily.^13^ However, it is important to note that comparing our study group to the critically ill ICU patients in Zander *et al*.’s study is challenging due to the complex and varying conditions experienced by ICU patients, such as multiorgan failure, capillary leak, hypoalbuminaemia and volume therapy. De Schepper *et al*. demonstrated that approximately 65% of piperacillin (60% of the total dose) is removed from serum during a 4‐h haemodialysis due to renal and biliary excretion.^14^ In their study, piperacillin was administered as a single 4000‐mg bolus injection over 3 min, resulting in mean concentrations of 110 mg/L before dialysis and 40 mg/L after dialysis.^14^ In contrast, our study found that 84% of piperacillin is removed during a 4‐h dialysis, with mean concentrations decreasing from 136.3 mg/L before dialysis to 22 mg/L after dialysis. However, the study by De Schepper *et al*. had a very small sample size (*n =* 3), which may have influenced their findings.^14^ Additionally, improvements in haemodialysis technology over the past 42 years could account for the differences in piperacillin removal rates. Similarly, our study included a small sample of haemodialysis patients (*n =* 10), so our results may not be generalizable to larger patient populations. Neither our study nor De Schepper *et al*.^14^ measured piperacillin concentrations in the dialysate, only in blood serum. Johnson *et al*. showed in their study in 1992 that during an approximately 3.2‐h haemodialysis, only 31% of piperacillin were removed from the blood.^15^ That number is even smaller than that observed by De Schepper *et al*.^14^ Johnson *et al*. recommends administering the usual dose not before but right after haemodialysis. That is also the procedure that was chosen for 7 of our 10 patients, who received their piperacillin dose right after and not before haemodialysis. In this study, we selected a conservative target of 60% for fT > MIC (16 mg/L). Additionally, we chose a more aggressive target of 100% fT > 4× MIC (16 mg/L).

Other common targets include 50% of the time or 100% of the time >MIC, and 50% of the time fT > 4× MIC.^13^, ^16^ However, these studies primarily address piperacillin concentrations in critically ill ICU patients, whereas our study focuses on noncritically ill patients from non‐ICU wards. Roberts *et al*. demonstrated that a positive clinical outcome was more likely with increasing 50% fT > MIC and 100% fT > MIC ratios for β‐lactams.^16^ With the more conservative target (60% of the time >MIC), PTA was >90% in all 4 groups of eGFR when being dosed as recommended by SmPC. Prolonged infusion of 3 h did increase the PTA in all 4 groups, but the PTA was relatively high even with short infusion. Our modelling using Monte Carlo simulations shows that the PTA reaches almost 100% in all groups (with the dosage interval as recommended by SmPC) when short infusions are switched to prolonged infusions over 3 h. Patients with eGFR <20 mL/min might profit from switching from 4.5 g q12‐h short infusions to 2.25 g q8‐h prolonged infusion. This regimen may result in higher PTAs, a lower risk of overdosing, and reduced antibiotic use. One can conclude that for the conservative target, dosing as recommended by SmPC is sufficient but not optimal for all patients. With the more aggressive target of 100% of the time >4× MIC, PTA was insufficient in all groups. Prolonged infusions over 4 h with an increased daily dose increased the PTA in all 5 groups, however, was also not adequate. Continuous infusions (daily dose was not higher than SmPC recommend, except for the group of eGFR 30 and 40 mL/min that received a daily dose of 16 g piperacillin instead of the recommended 12 g daily) clearly resulted in the highest PTAs (>90% in all groups). Continuous infusions should ideally be administered in conjunction with therapeutic drug monitoring (TDM) to prevent under‐ or overdosing. One can conclude that for the more aggressive target, dosing as recommended by SmPC is insufficient. While some find TDM unnecessary for β‐lactams, as their therapeutic range is not as narrow as that of for example aminoglycosides, 2 randomized controlled trials have found that TDM improves the achievement of PK/PD targets.^17^, ^18^, ^19^ It has to be noted increased attainment of PK/PD targets does not generally imply positive clinical outcome.^20^

Patients in the 2 randomized controlled trials have differed from our group of patients, as they explored β‐lactam concentrations in patients with neutropenic fever and in ICU patients, in contrast to our group of non‐ICU patients with decreased renal function. MIC > 16 mg/L is a pretty conservative aim and other studies exploring piperacillin PK in critically ill patients aim for a MIC > 16 mg/L for 100% of the dosage interval or even MIC > 4× 16 mg/L for 50% of the time.^13^ We decided on an aggressive target of fT > 4× MIC, as a French study suggests this PK/PD target, even though they only refer to ICU patients and our group of patients consists of non‐ICU patients.^7^ They also suggest prolonged or continuous infusions when infections are caused by bacteria with a high MIC (e.g. *P. aeruginosa*). PK parameters obtained from the modelling process were highly consistent with PK parameters reported in the literature: in this work, we found a typical piperacillin clearance of 2.3–4.9 L/h for nondialysis patients with an eGFR of 10 mL/min up to 40 mL/min, respectively. The median population clearance found in the literature is 2.76 L/h (range: 1.4–7.9 L/h) and the reported value is in accordance to other population PK analysis on comparable adult populations.^2^, ^21^, ^22^, ^23^, ^24^, ^25^ The population estimate for volume of distribution of the herein presented model is 15.4 L for an adult patient, whereas literature values range from 10 to 120 L. However, most of the more recent results are found at the lower end of this range^2^, ^22^, ^23^, ^24^, ^25^

There are limitations to the study. (i) Creatinine clearance was not calculated, as it is not regularly determined in non‐ICU patients. Instead, we used the estimated GFR (eGFR) as calculated by the central laboratory at both hospitals. (ii) Pathogens for which the patients received PTZ were not collected for this study. Therefore, we used the MIC for unknown pathogens given by EUCAST set at 16 mg/L for PTZ.^6^ (iii) Liver function was not noted for the patients and decreased liver function was not an exclusion criteria, even though studies show that a significant amount of piperacillin is excreted via the biliary system.^14^, ^26^, ^27^ (iv) The sample size in this study was rather small, especially for group 3 which consisted of only 10 patients.

There are also some strengths in the study. (i) There was a wide heterogeneity of patient characteristics: Patients had a big variety of renal impairment (moderate and severe renal impairment as well as patients undergoing iHD), ages ranging from 35 to 88 years and BMIs from <18.5 kg/m^2^ to >40 kg/m^2^. (ii) We used Monte Carlo simulation to apply our results to a broader group of patients.^9^ (iii) The clinical outcome was not subject of this study. Switching from short infusions to continuous infusions for patients with renal impairment might attain the more aggressive PK/PD target when an infection with a pathogen classified as *susceptible, increased exposure (I)* is proven or suspected.

While dosing as recommended by SmPC seems to be sufficient for the conservative PK/PD target, it is insufficient when aiming a more aggressive PK/PD target. Although there is currently neither evidence nor recommendations to aiming for a more aggressive target in non‐ICU patients, clinicians may tend to increase their aimed target when dealing with severe infections in difficult to penetrate foci caused by *P. aeruginosa*. Further research could investigate whether non‐ICU patients would benefit from a more aggressive strategy. While we examined the concentrations achieved by patients with renal impairment and adapted doses of PTZ, it would be interesting to identify other groups of noncritically ill patients at risk of failing to meet PK/PD targets. Additional studies should also determine if there are any drawbacks to continuous infusions and whether risks exist when transitioning from short to prolonged or continuous infusions in this patient population.

Administering 4.5 g of PTZ every 8 or 12 h via short infusions may result in insufficient piperacillin concentrations in a significant number of patients if a pathogen is proven or suspected to be *susceptible, increased exposure (I)* and an aggressive PK/PD target is set. Switching to continuous infusions increases the PTA of 100% of the time >4× MIC, so it may be prudent to deliver PTZ continuously even for patients with decreased renal function in this discussed setting. The feasibility of a continuous or even prolonged administration of piperacillin bears frequent difficulties, irrespective of the nature of the wards to which patients are submitted. Several patients have scheduled examinations or interventions that not only intervene with the administration time. Implementing standard operation procedures instructing on the most acceptable disconnected infusion time or listing criteria for most favourable settings (e.g. surgery) *vs*. concurrent challenges (e.g. haemodialysis) may be helpful in considering the modus operandi. Our study does not provide final answers for patients on piperacillin for suspected or identified pathogens wanting an increased exposure, and we do lack the comparison of more clinical data to our results, but it may fuel the debate and present guidance not only for EUCAST but for a more distinct clinical application too. In the meantime, EUCAST has established a working group to develop recommendations for this exact topic.

## AUTHOR CONTRIBUTIONS

E.D., S.H., P.S., M.K., O.S.C. and G.S. participated in research design and revision of the manuscript. E.D. conducted, analysed the data and wrote the manuscript. S.H. provided samples from Jena and participated in the analysis of the data. P.S. conducted and analysed the data in Jena. M.K. processed the samples, analysed the data and revised the manuscript. O.S.C. conducted the main analyses, supervised and revised the manuscript. G.S. coordinated, supervised and wrote the manuscript. All authors read and approved the final manuscript.

## CONFLICT OF INTEREST STATEMENT

None.

## Supporting information


**TABLE S1** Model development.
**FIGURE S1** Observed piperacillin plasma concentrations *vs.* model predicted concentrations.
**FIGURE S2** Scatterplots of individual weighted residuals (IWRES) *vs.* time and *vs.* concentration. Individual parameters are obtained as empirical bayes estimates (mode of the conditional distribution). The blue line represents the least squares linear regression of IWRES with the 95% confidence interval (shaded area).
**FIGURE S3** NPDE plots. NPDE, normalized prediction distribution error.
**FIGURE S4** eGFR and piperacillin clearance.

## Data Availability

The data that support the findings of this study are available on request from the corresponding author. The data are not publicly available due to privacy restriction.
