# Supplementary material for: Probability of pharmacokinetic/pharmacodynamic target attainment for different piperacillin/tazobactam dosing regimens in renally impaired patients in a non‐intensive care unit setting
Source: Br J Clin Pharmacol. 2025 Jun 29;91(11):3070–81. doi: 10.1002/bcp.70153 (PMC12569551; doi:10.1002/bcp.70153)
Supplement: Supplementary file 1 — TABLE S1 Model development. FIGURE S1 Observed piperacillin plasma concentrations vs. model predicted concentrations. FIGURE S2 Scatterplots of individual weighted residuals (IWRES) vs. time and vs. concentration. Individual parameters are obtained as empirical bayes estimates (mode of the conditional distribution). The blue line represents the least squares linear regression of IWRES with the 95% confidence interval (shaded area). FIGURE S3 NPDE plots. NPDE, normalized prediction distribution error. FIGURE S4 eGFR and piperacillin clearance. [file BCP-91-3070-s001.docx]

**Electronic Supplement**

| **Model** | **-2 LogLikelihood** | **Corrected BIC** | **Error model** | **Covariates** |
| --- | --- | --- | --- | --- |
| BaseModel 1 | 1338.4 | 1370.63 | Combined 1 | - |
| BaseModel 2 | 1338.19 | 1370.43 | Combined 2 | - |
| BaseModel 3 | 1421.07 | 1448.42 | Constant | - |
| BaseModel 4 | 1342.94 | 1370.28 | Proportional | - |
| CovariateModel 1 | 1323.91 | 1355.15 | Proportional | eGFR on CL |
| CovariateModel 2 | 1314.43 | 1349.56 | Proportional | eGFR on CL  BSA on Vd |

**Supplement Table 1:** Model development

| 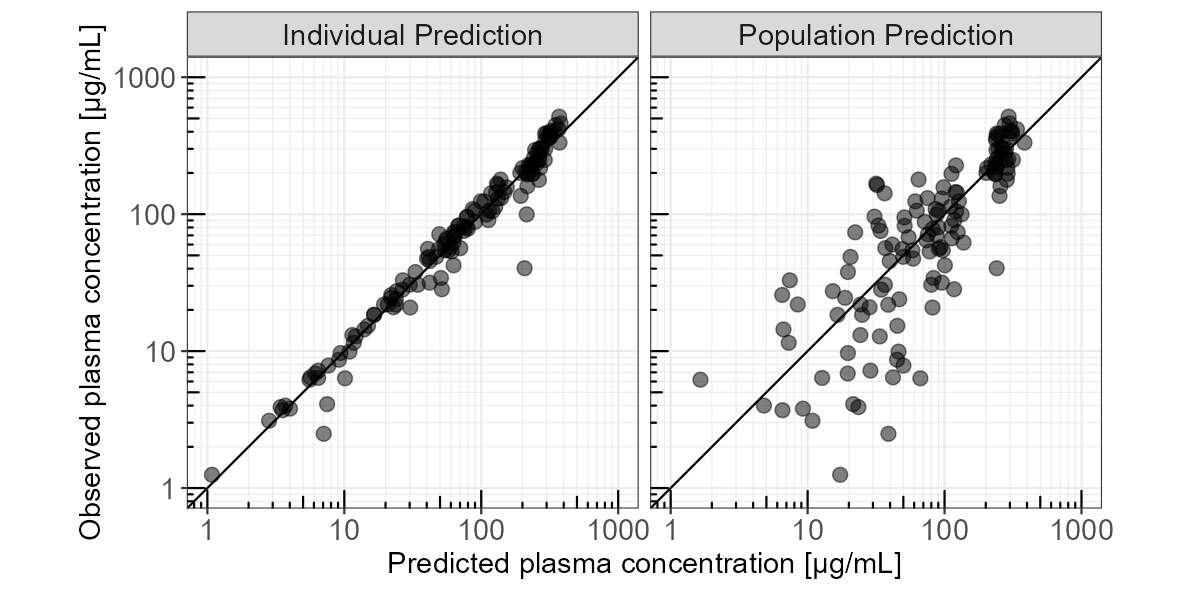 |
| --- |
| **Supplement Figure 1:** Observed piperacillin plasma concentrations vs. model predicted concentrations. |

| 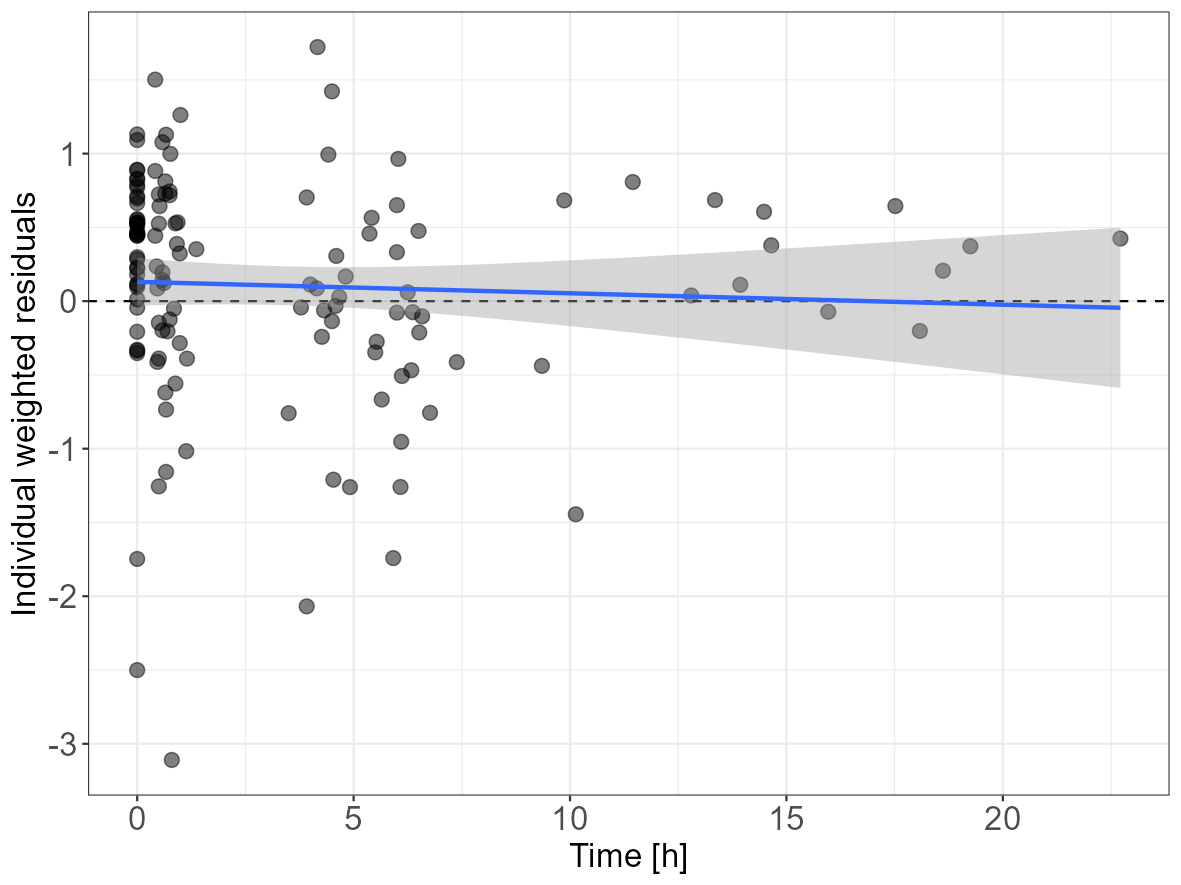  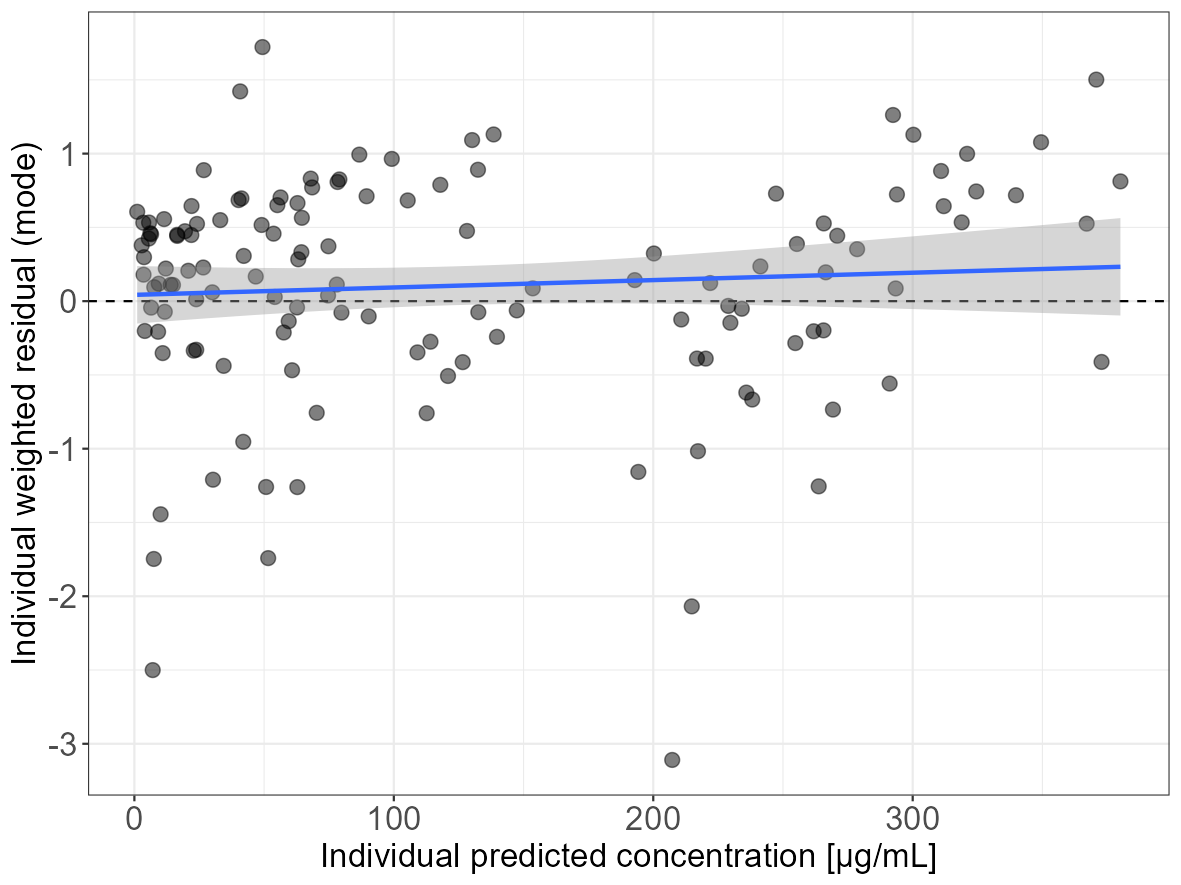 |
| --- |
| **Supplement Figure 2:** Scatterplots of individual weighted residuals (IWRES) vs. time and vs. concentration. Individual parameters are obtained as empirical bayes estimates (mode of the conditional distribution). The blue line represents the least squares linear regression of IWRES with the 95% confidence interval (shaded area). |


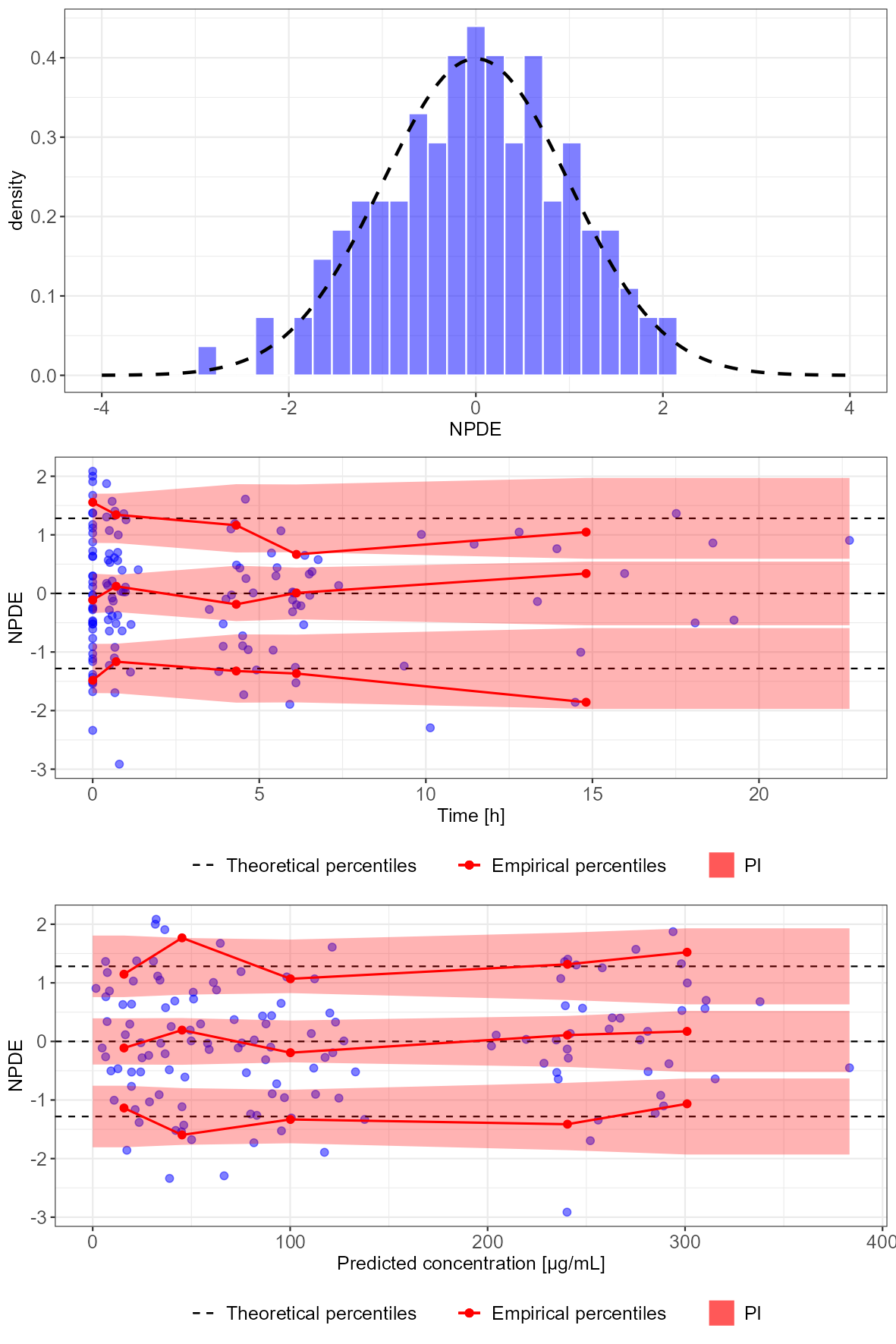


**Supplement Figure 3:** NPDE plots. NPDE, normalized prediction distribution error.

**Supplement Figure 4:** eGFR and piperacillin clearance
